# Larval Host Preference and Suitability for the Sawfly *Mesoneura rufonota* among Five Cinnamomun Tree Species

**DOI:** 10.3390/insects11020076

**Published:** 2020-01-22

**Authors:** Jiaying Zhou, Jiangtao Zhang, Tom Tregenza, Youliang Pan, Qinzhao Wang, Haoni Shi, Xingping Liu

**Affiliations:** 1 Key Laboratory of State Forestry and Grassland Administration on Forest Ecosystem Protection and Restoration in Poyang Lake Watershed, College of Forestry, Jiangxi Agriculture University, Nanchang 330045, China; jyzhou1106@outlook.com (J.Z.); jiang_tao_zhang@163.com (J.Z.); pan1649034597@163.com (Y.P.); qinzhaowang@163.com (Q.W.);; 2 Centre for Ecology & Conservation, School of Biosciences, University of Exeter, Penryn Campus, Falmouth, Cornwall TR10 9FE, UK; T.Tregenza@exeter.ac.uk

**Keywords:** *Mesoneura rufonota*, host suitability, host preference, performance, *Cinnamomum*

## Abstract

The camphor sawfly, *Mesoneura rufonota* Rohwer, is an economically important leaf-chewing pest of the genus *Cinnamomum Trew*. However, little is known about the range of species that it can infest within this genus or whether larvae show preferences for particular species. We conducted preference and performance experiments under laboratory conditions to assess larval host preference and suitability as a host plant of five congeneric trees species: *C. camphora* (Linn) Presl, *C. bodinieri* Levl., *C. burmanni* (Nees et T. Nees) Blume, *C. pauciflorum* Nees, and *C. micranthum* (Hay.) Hay. In no-choice, two-choice and multiple-choice feeding trials, significantly higher feeding rates were found on *C. camphora*, followed by *C. bodinieri*, compared to those on the other three tree species. In two-choice behavior trials, larvae moved to occupy leaves of *C. camphora* faster and more frequently, followed by *C. bodinieri*, than when offered the other three tree species. In no-choice fitness trials, the survival of larval and pupal stage was the highest, the developmental duration of larval and pupal stage was the shortest, the pupal body weight was the heaviest and adults lived the longest on *C. camphora* followed by *C. bodinieri*, while *M. rufonota* did not complete development on *C. burmanni*, *C. pauciflorum* or *C. micranthum*. Our results demonstrate that larvae consistently prefer and perform better on *C. camphora* leaves, that they can utilize *C. bodinieri*, but it is less preferred, and that *C. burmanni*, *C. pauciflorum,* and *C. micranthum* appear to be unsuitable as a host for *M. rufonota*.

## 1. Introduction

Insects constitute the most diverse group of animals on Earth, and a large fraction of insect species are phytophagous [1]. Exploring the relationship between phytophagous insects and host plants has been a central topic for many evolutionary ecologists, biologists, and pest control experts [2,3,4]. Host plant range is a key ecological characteristic for phytophagous species as it defines their resource base, which in turn is an important factor influencing their population dynamics and interactions with other phytophagous species, predators, and parasites [5]. Thus, it is of great significance in evolutionary ecology theory and pest control practice to investigate the host plant range of phytophagous insects. In nature, phytophagous insects differ in their degree of specialization on host plants, and range from strictly monophagous species to extremely polyphagous species [6,7,8]. For oligophagous and polyphagous insects, the selection of a suitable host plant is essential to ensure their development and reproduction [3,9], and having a large host plant range is considered to be an evolutionary advantage [10]. A broad host range may allow oligophagous species to colonize new hosts in areas where their original or preferred hosts are absent or much less abundant [11]. Understanding the process of host plant selection by phytophagous insects and their impact on various life-history traits has been a major goal of insect evolutionary ecology [12,13]. In recent decades, the behavioral mechanisms by which phytophagous insects locate and select potential host plants have been extensively documented [14,15]. However, the vast majority of this work has focused on host plant selection of phytophagous insects for feeding or egg-laying by adult insects [3,7,16,17,18], while the role of larval stages in host plant selection has received less attention in empirical studies to date [19,20,21,22].

The genus *Cinnamomum Trew* belonging to the family Lauraceae comprises about 250 species which are distributed in China, India, Sri-lanka, and Australia [23]. Forty-six species are native to China and some of them are widely cultivated in southern China as shade trees because they are fragrant evergreen broad-leaved species, such as *Cinnamomum camphora* (Linn) Presl, *C. bodinieri* Levl., and *C. micranthum* (Hay.) Hay etc. They are also economically important for medicine, pesticides, natural flavors, and fragrances [24]. However, in recent years, a large number of pests in these trees have occurred with the gradual enlargement of the cultivation area. The camphor sawfly, *Mesoneura rufonota* Rohwer (Hymenoptera: Tenthredinidae), is an economically important leaf-chewing insect pest, native to China and first reported in the 1960s on camphor tree *C. camphora* [25]. Previous studies of the biological characteristics indicated that this pest can complete one to seven generations per year in different regions in China and overwinters as mature larvae (prepupae) within cocoons in the soil [26,27,28]. Adults usually leave their pupation cells in mid to late March. Newly emerged adults exhibit stronger flight ability. Females lay eggs on the tender leaves of host plants, beginning shortly after leaving their pupal chambers. The larvae then feed gregariously on the tender leaves, causing large economic losses and ecological effects [26]. The larvae are considered to be oligophagous. Their reported host plants focus on the genus *Cinnamomum* and the primary host is *C. camphora* [25,26]. Larvae often move frequently to forage host plants under field conditions, especially in the case of lacking food. Situations where two host plants of different species are in contact with one another such that larvae may need to choose between the two species, will not be very common in nature but they will occasionally occur. Some interesting questions arise from here. Are there alternative host plants that might allow populations that would otherwise be inviable to survive in areas where the primary host is less common? Could alternative hosts allow *M. rufonota* to spread across areas where the primary host is absent? Alternatively, are there other plant species that sawfly larvae will attempt to feed on, but where they cannot complete development? Such species could potentially be useful in control strategies.

Considering the potential for this pest to cause heavy damage and the economic importance of the genus *Cinnamomum*, there is a critical need for broader and more precise information on host preference and suitability of this pest. This information is indispensable to better understand the potential effect of the camphor sawfly on different host species. However, currently, there is no information available in the literature on the host preference of this pest among different species of the genus *Cinnamomum*. To our knowledge, no attempt has been made to investigate the life history traits of *M. rufonota* on the different species of the genus *Cinnamomum* in China. Therefore, we sought to determine the larval preference and performance for five congeneric tree species of the genus *Cinnamomum* commonly present in China.

Our first objective was to compare the leaf-feeding preference and rate of larvae among five different plant species using no-choice, two-choice, and multiple-choice feeding trials. Our second objective was to assess the extent to which larvae would choose to move towards and begin feeding on plants of different species through two-choice trials. Our third objective was to determine the impact on several life-history parameters of this insect of different potential host plants. The results of our study will throw light on the potential host plant range of this sawfly with potential applications for integrated pest management.

## 2. Materials and Methods

### 2.1. Plant Materials

To represent a range of the most common potential host species, we collected five congeneric tree species in the genus *Cinnamomum* which are widely cultivated in the landscape in China as a subset of the potential host plants to use in this study: *C. camphora* (Linn) Presl (CCP), *C. bodinieri* Levl. (CBL), *C. micranthum* (Hay.) Hay (CMH), *C. burmanni* (Nees et T. Nees) Blume (CBB), and *C. pauciflorum* Nees (CPN). These trees were cultivated in the nursery garden of Jiangxi Agricultural University. Freshly tender leaves were collected from different individuals of each species and used for the following experiments.

### 2.2. Insects Rearing

All *M. rufonota* larvae used in the experiments were obtained from the second generation of a colony originally derived from natural populations of multiple broods in Jinxi County, Jiangxi Province, China (28°2′33″ N, 116°44′39″ E) in March 2019. Third to fourth instar larvae were collected from the tender leaves of *C. campora* and brought into the forest protection laboratory of Jiangxi Agricultural University. The wild-collected larvae were placed in transparent plastic boxes (length × width × height = 20 × 15 × 7 cm) and maintained in artificial climate incubators at a constant temperature of 25 ± 1 °C and 70 ± 10% relative humidity with 14 h light: 10 h dark regime. To reduce maternal effects and to expose the larvae to all the new hosts before the commencement of the experiments, we reared larvae with freshly collected tender leaves from a mixture of all five potential host plants simultaneously. Host plants were provided in excess and replaced daily until the pre-pupal stage was attained. Upon maturation, we allowed larvae to pupate and placed pupae into empty wooden mesh cages (length × width × height = 30 × 30 × 40 cm). Newly emerged adults were transferred into new cages and permitted to mate freely. They were provided with a fresh branch of camphor tree as mating and oviposition site. Newly laid eggs were collected daily and transferred into transparent plastic boxes lined with moistened filter paper and placed in incubators under the same conditions. Standard camphor sawfly rearing techniques were used throughout the study [28]. Newly hatched larvae were used in the larval performance experiment or were colony reared using the same method described above until their third instar. Then the third-instar larvae were used in larval preference experiments. We conducted all of these experiments at 25 ± 1 °C and 70 ± 10% relative humidity with 14 h light: 10 h dark regime in artificial climate incubators (larval performance experiments) and air-conditioned chamber (larval preference experiments). These conditions represent a subset of the conditions that host and insects experience in the field.

### 2.3. Larval Preference Experiments

#### 2.3.1. Larval Feeding Rate across Host Plants

To examine larval feeding rates on different host species, we performed no-choice, two-choice and multiple-choice leaf-feeding trials simultaneously. Leaf squares (length × width = 2 × 2 cm) were cut from the freshly harvested tender leaves of each of the five plant species before the experiments were conducted. In no-choice feeding trials, a leaf square of one of the five potential host plants was placed in the center of a small plastic petri dish (d = 8.5 cm, h = 1.5 cm) lined with moist filter paper. In two-choice feeding trials, two leaf squares of two different plant species were placed in a similar petri dish. In multiple-choice feeding trials, leaf squares from each of the 5 plants species were arranged at random, avoiding overlapping, in a large plastic petri dish (d = 20 cm, h = 2.5 cm). A single third-instar larva that had been kept isolated from food for the previous 3 h was transferred into the dish. All of the petri dishes were sealed with thin transparent plastic wrap to prevent larva escape. After 24 h, traces of larval feeding were observed and area (%) of the leaf square eaten was calculated. Leaf area consumed was measured by placing the consumed leaf squares on top of 1-mm-grid graph paper. The leaf was carefully outlined with a pencil on the graph paper. The total number of grid squares within the outline of the leaf was counted. The leaf squares with the largest and smallest area eaten represented the most and least preferred plants for larval feeding, respectively. Each possible host plant or combination of host plants was replicated thirty times in each design.

#### 2.3.2. Larval Choice Behavior across Host Plants

To compare the attractiveness of different host plants, two-choice behavior trials were performed using leaf squares. We placed two freshly cut 4 cm^2^ leaf squares derived from different plant species in a small plastic petri dish lined with moist filter paper. A single third-instar larva that had been food-deprived for the previous 3 h was transferred into the center of the petri dish. We observed the dish continuously and counted the larvae on each particular tested leaf square, and recorded the time between introduction and the first observation of feeding. Observations were continued for 1 h. Thirty replicates of each of the 10 possible pairwise combinations of five host plants were performed. Larvae that did not feed at any point during the observation were discarded from the analysis.

### 2.4. Larval Performance Experiments

To determine whether plant species affect larval performance, we compared life-history parameters among newly hatched larvae reared on each of the five potential host plants. We performed 10 replicates of our life-history study for each tree species. Within each replicate, we selected thirty newly hatched larvae randomly and transferred them into a transparent rectangular plastic box (length × width × height = 20 × 15 × 7 cm) lined with moist filter paper and containing excess freshly harvested tender leaves from one of the five host plants. We replaced the filter paper and food daily and monitored larvae until pupation. We recorded the developmental duration of the larval stage, the number of larvae surviving to pupation and the weight of the pupa. We harvested and placed cocoons into a small transparent plastic cylinder (d = 5 cm, h = 10 cm) using soft forceps. When adults emerged from their cocoons, we recorded the developmental duration of the pupal stage and counted the number of adults as measures of the survival rates of the pupal stage. On emergence, sawflies were sexed and placed individually in small transparent plastic cylinders. We recorded adult lifespan in the absence of host species.

### 2.5. Statistical Analysis

All data analyses were carried out with SPSS version 19.0 for Windows (SPSS Inc., Chicago, IL, USA). The proportion data were arcsine square-root transformed prior to analysis. Then the data were first checked for homogeneity of variance and normality using frequency histograms and the Shapiro-Wilks test. Non-parametric tests were performed in cases where variances were heteroscedastic and/or distributions were not normal. We performed Kruskal–Wallis tests in no-choice trials and in multiple-choice trials, and independent sample Mann–Whitney U-tests in two-choice trials to explore the effects of tree species on larval feeding rate and the time taken to move to the leaf. We performed a binomial test to compare the difference in terms of the numbers of larvae approaching each species in two-choice trials. Life history parameters were analyzed using one-way ANOVA. Although survival itself is a dichotomous trait, the distribution of the proportion of individuals surviving within each host plant was better suited to a normal distribution according to our experiment design. The least significant difference (LSD) method was used for comparison of means. Data are expressed as median and interquartile 25–75% range in the larval preference experiment and are presented as means ± SE in the larval performance experiment.

## 3. Results

### 3.1. Larval Feeding Rate across Host Plants

**Figure 1:**
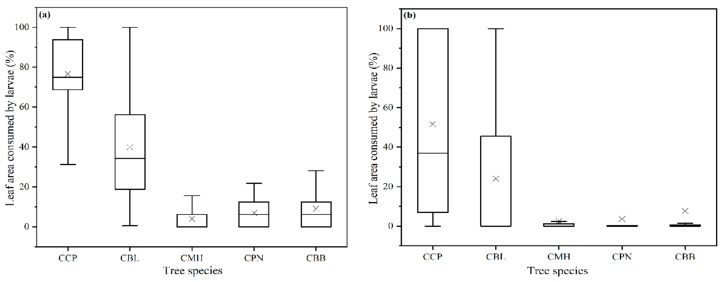
Median percentage (with interquartile 25–75% range in parentheses) of leaf area consumed by *Mesoneura rufonota* larvae under (**a**) no-choice trials and (**b**) multiple-choice trials. (CCP: *C. camphora* (Linn) Presl, CBL: *C. bodinieri* Levl., CMH: *C. micranthum* (Hay.) Hay, CBB: *C. burmanni* (Nees et T. Nees) Blume, CPN: *C. pauciflorum* Nees). The line and crosses inside the boxes indicate the medians and means, respectively; the heights of the boxes indicate the first and third quartiles, and the whiskers indicate the data range.

All of the five tested tree species were eaten by *M. rufonota* larvae to some extent, although the extent of their utilization as a host plant varied substantially. When larvae were restricted to feeding on one of the tree species in no-choice trials, larvae fed on significantly larger portions of CCP and CBL than on the other tree species with the feeding rate of 75% and 34%, respectively. The feeding rate per larva varied significantly among tree species tested (Kruskal-Wallis test: *H* = 102.97, *df* = 4, *p* < 0.001; Figure 1a). However, no significant difference was found in the feeding rate when larvae were restricted to feeding on CMH, CPN, and CBB (*p* > 0.05). When given a choice of all five tree leaves together in multiple-choice trials, a similar trend was observed with lower feeding rates on CBL than CCP and very low incidence of feeding on CMH, CPN, and CBB (Kruskal-Wallis test: *H* = 35.99, *df* = 4, *p* < 0.001; Figure 1b). In addition, when given a choice between pairs of tree species in two-choice trials, larvae significantly favored CCP, followed by CBL, with CBB, CPN, and CMH in low-ranking positions (Mann-Whitney U-test, *p* < 0.001), except for in the pairwise combination of CPN and CBB (Mann-Whitney U-test, *W* = 368, *p* = 0.22, Table 1).

### 3.2. Larval Choice Behavior across Host Plants

**Figure 2:**
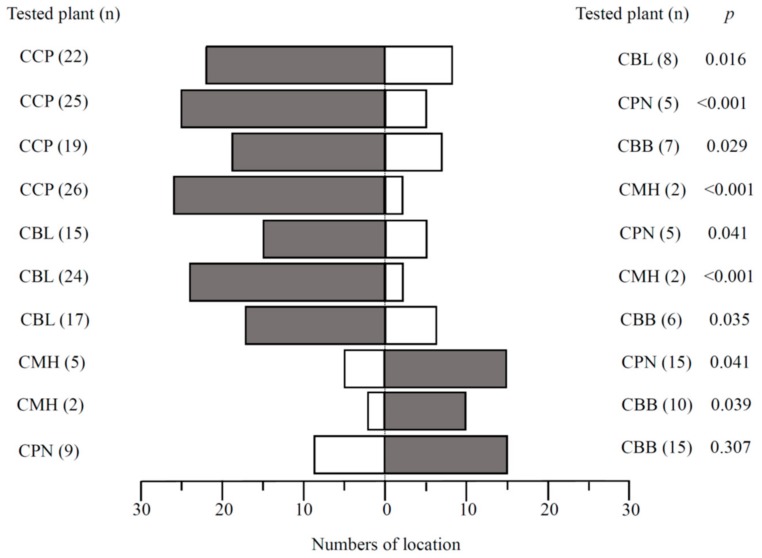
Numbers of *Mesoneura rufonota* larvae on leaves of each tree species in 10 pairwise combinations in two-choice trials.

**Figure 3:**
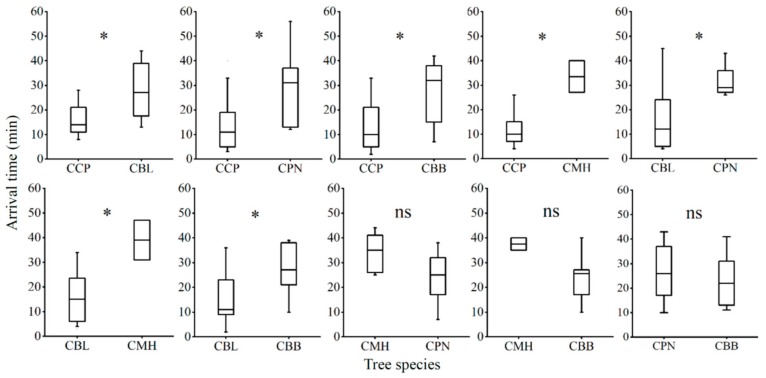
Time it took *Mesoneura rufonota* larvae to move to a leaf square of each tree species in 10 pairwise combinations in two-choice trials (* 0.01 < *p* < 0.05, ns = not significant (*p* > 0.05)).

Although there were some larvae that did not approach a leaf, apart from the combinations CCP vs. CBL or CPN, third-instar larvae did move to occupy a leaf from one of the species offered in the two-choice leaf square trials. Significantly more larvae moved to CCP than CBL, and relatively few larvae were found on CMH than on the other tree species in its pairwise combinations (Binominal test, *p* < 0.05, Figure 2). The time it took larvae to crawl to the different tree species was assessed among ten possible plant combinations. When CCP was combined with other tree species, larvae found their way to CCP significantly faster than to the other tree species (Mann-Whitney U-tests: CCP vs. CBL: *W* = 35.5, *p* = 0.012; CCP vs. CPN: *W* = 23.5, *p* = 0.027; CCP vs. CBB: *W* = 25.5, *p* = 0.015; CPP vs. CMH: *W* = 1, *p* = 0.011; Figure 3). CBL was also more attractive to larvae than CBB, CPN, and CMH (Mann-Whitney U-tests: CBL vs. CPN: *W* = 10.50, *p* = 0.015; CBL vs. CBB: *W* = 24.5, *p* = 0.028; CBL vs. CMH: *W* = 3, *p* = 0.037; Figure 3). However, no statistical difference was evident across CBB, CPN, and CMH leaves in their pairwise combinations (Mann-Whitney U-test: *p* > 0.05, Figure 3).

### 3.3. Larval Performance across Host Plants

Host plant-related life-history traits of *M. rufonota* are presented in Table 2. There was an 84% survival rate for larvae reared on CCP, followed by 45% for CBL, whereas less than 10% of larvae reared on CMH, CPN, and CBB survived. The differences were significant (ANOVA, *F* = 249.24, *df* = 4, 45, *p* < 0.0001). The duration of larval development was the shortest on CCP and longest on CMH. There were significant differences in larval developmental durations among the five tree species (*F* = 4.47, *df* = 4, 466, *p* = 0.001). The food consumed by *M. rufonota* during its larval stage significantly affected pupal weight, with heavier pupal weight on CCP than on other tree species (*F* = 9.16, *df* = 4, 129, *p* < 0.0001). No adults eclosed from CMH, CPN, and CBB food treatments. Significantly higher pupal survival, shorter developmental duration in pupal stages, and longer adult lifespan was observed in the CCP treatment compared to the CBL treatment (pupal survival: *F* = 341.25, *df* = 4, 45, *p* < 0.0001; pupal developmental duration: *F* = 19.74, *df* = 1, 295, *p* < 0.0001; adult lifespan: *F* = 11.47, *df* = 1, 295, *p* = 0.001).

## 4. Discussion

Three important findings arise from our study. First, *M. rufonota* larvae exhibited significant host preferences among the five congeneric tree species we provided. Larvae preferred *C. camphora*, followed by *C. bodinieri*, to *C. burmanni*, *C. pauciflorum,* and *C. micranthum*. Second, more *M. rufonota* larvae moved towards and onto *C. camphora* leaf squares and they did so more rapidly than on the other tree species. Finally, *M. rufonota* larvae had dramatically higher performance in terms of development and survival of immature stages, pupal weight, and adult lifespan when reared on *C. camphora*, and did not complete development on *C. burmanni*, *C. pauciflorum*, and *C. micranthum* as manifested by higher larval and pupal mortality. Assuming that shorter development times, higher survival, and higher final mass are advantageous (as seems likely), these results demonstrate that *C. camphora* is the most preferred and suitable host for *M. rufonota* larvae. *C. bodinieri* is a potentially suitable host and *C. burmanni*, *C. pauciflorum*, and *C. micranthum* appear to be unsuitable as a host for *M. rufonota*.

The frequency and suitability of host plant species encountered by phytophagous insects can vary in time and space because of heterogeneity in the environment, disturbance, colonization, and intra- or interspecific interactions [29]. For herbivorous insects, ecological theory and empirical data on host plant selection provide evidence that adults can engage in host-plant selection for their offspring by choosing to lay their eggs on particular plants [3,7,13,17,18,29,30,31], However, there is now accumulating evidence revealing that immature life stages can also play an active role in host discrimination, especially in Lepidopterans [19,20,21,22,32,33]. We find evidence of *M. rufonota* larval preference for *C. camphora* and *C. bodinieri* as measured by the mass of leaf consumed by the larvae and by the larval choice of host plants and time spent on each leaf. Our findings provide a new example regarding interactions between host selection behaviors and plant preferences of an oligophagous Hymenopteran.

When leaf squares were presented individually or in multiples, we found examples of larvae feeding on all of the species we tested (Figure 1). This was expected since the threat of starvation will drive many insects to exhibit wider polyphagy when they only have access to less-suitable host plants [34]. Similar behavior has been observed in other insects, such as *Trichoplusia ni* [32]. However, when *M. rufonota* larvae were offered pairwise combinations of host plants, they showed a clear preference for *C. camphora*, followed by *C. bodinieri*, with *C. burmanni*, *C. pauciflorum*, and *C. micranthum* in low-ranking positions, indicating that *M. rufonota* larvae have evolved the capacity to detect and favor their main natural host plants (Table 1, Figure 2 and Figure 3). The mechanisms underlying potential host selection by *M. rufonota* larvae are still uncertain. The superficial similarity of the leaves of the five species, particularly once they are cut into square pieces makes visual discrimination unlikely. It is likely that olfactory signals originating from host plants are used by larvae as reported in a number of phytophagous insects [12,35,36], providing evidence that olfaction can mediate orientation in immature stages [20].

Numerous studies have reported that the biological and life history parameters of many insects, such as development, survival, body weight and reproductive rate, differ significantly among host species [37,38,39] or cultivars [40]. These effects on life history are important determinants of plant suitability for phytophagous insects. Shorter development times, higher survival, and higher rates of reproduction of insects on a host indicate greater suitability [41,42]. Our results demonstrate a positive correlation between larval preference and their performance on the plants that we provided to them. Larvae in non-choice performance assays were more likely to complete their development on the species that were most preferred in our preference assays.

Developmental duration is potentially an important component of fitness, as it determines how long different pre-reproductive stages are exposed to hazards including predators and parasites [38]. Our no-choice fitness trials show that the developmental duration of larval and pupal stages of camphor sawfly was remarkably shorter on *C. camphora* than on other host species (Table 2). The shorter developmental duration may provide an important selective advantage under pressure from natural enemies, as demonstrated by several authors [41]. Thus, a reduction in developmental duration on *C. camphora* could represent an advantage to *M. rufonota* by reducing its vulnerability to parasitism and predation. Similar results have been reported in other insect species, such as *Plutella xylostella* [42]. Although development was relatively poor on *C. bodinieri*, it also supported the development and population establishment of *M. rufonota*.

Our results revealed distinct differences in survival of *M. rufonota* immature stages, with the sawfly failing to complete development on *C. burmanni*, *C. pauciflorum*, and *C. micranthum*. Therefore, we expect these tree species will have lower infestations in the field due to their lower suitability. Previous field studies have suggested that *C. camphora* is the plant most heavily infested by *M. rufonota* [26,28]. Our results concur with these findings as well as providing evidence that *C. bodinieri* is a potentially suitable host for the survival of *M. rufonota*. *M. rufonota* performed the worst on *C. burmanni*, *C. pauciflorum*, and *C. micranthum* as manifested by higher larval and pupal mortality (Table 2). Numerous studies in phytophagous insects have demonstrated that larger pupal size is beneficial for subsequent reproduction, showing the positive relationship between pupal mass and adult fecundity [41]. Heavier pupal weight was found in *C. camphora* reared larvae in our experiment, further showing *C. camphora* is the dominant host plant for *M. rufonota*. In many Hymenopteran, adults utilize nutrients derived from larval reserves for maintenance as they do not feed [43]. Thus, adult lifespan is dependent entirely on resources acquired during larval development [44]. Our results show that the adult lifespan was significantly longer on *C. camphora* than on *C. bodinieri* (Table 2), also suggesting *C. camphora* is a more suitable host to *C. bodinieri* for *M. rufonota*.

We did not specifically control the diet of the mothers of insects used in our study so theoretically, non-genetic maternal effects could have influenced the host plant preference of the individuals we studied. There is very limited evidence for strong maternal effects on host plant preferences in insects so we regard this as unlikely to be a part of the explanation for our results. A related issue is that although we provided larvae pre-feeding-trials with a mixture of food plants, they are likely to have eaten more of some species than other species. If larvae have some retained preference for host plants that they have fed on previously, this could affect their subsequent preferences. Investigating this would need further experiments.

In general, our findings reveal differences in the performance of lab-reared *M. rufonota* larvae on five potential host trees. Of the five host species tested, *C. camphora* exhibited high-quality host, followed by *C. bodinieri*, and *C. burmanni*, *C. pauciflorum*, and *C. micranthum* being consistently the least suitable host. There are three probable reasons to explain these results. First, some constituent compounds, especially nutrients, acquired from the host plants the individuals feed on can greatly impact the performance and fitness of individuals [45]. Second, plant primary and secondary metabolites or toxic compounds may have a strong influence on the performance of phytophagous insects [39,46,47,48]. Finally, physiological barriers inherent in these host plant species may affect feeding, and consequently, led to differences in the development and survival of insects [49]. Concerning *M. rufonota* larvae, secondary plant substances or physiological barriers seem to be the factors that play a decisive role in its feeding and growth, as closely related plant species may be expected to yield similar nutritional value to feeding herbivore [39]. However, the exact cause of the differences found among host plants in larval host preferences and performance remains unknown and, therefore, additional research is needed to biochemically establish the reasons for the differences.

## 5. Conclusions

Our study reveals that at least one other member of the genus *Cinnamomum* is both attractive to *M. rufonota* larvae and allows them to complete development. This suggests that it would be worth testing other species that we did not include in the present study. Our findings that three other common *Cinnamomum* species have very low attractiveness for larvae and are not suitable host plants for their development indicates that these species might be planted in areas where outbreaks of *M. rufonota* are a problem. It also suggests that these species are unlikely to allow the spread of *M. rufonota* through areas with a very low density of suitable hosts. The possibility that we might find a species among the five we examined that is attractive to larvae but which they cannot actually utilize as a host-plant was rejected. Our finding that larvae have evolved the capacity to identify suitable hosts using airborne chemical cues and move towards them suggests that it might be possible to develop control methods for *M. rufonota* which utilizes this chemotactic response. Further work to investigate the oviposition preferences of *M. rufonota* adults in relation to potential host plants would provide valuable complementary information to that offered in our study.

## Figures and Tables

**Table 1:** Median percentage (with interquartile 25–75% range in parentheses) of leaf squares (cm^2^) consumed by *Mesoneura rufonota* larvae under 10 pairwise combinations in two-choice trial.

Tree Species		CCP	CBL	CMH	CPN
CBL	Percentage	97.9 (71.8, 100); 63.9 (30.1, 76.1)			
	*W*	61.0 **			
CMH	Percentage	100 (100, 100); 0 (0, 0.3)	91.5 (78.1, 95.6); 0 (0, 0.1)		
	*W*	0 **	9.5 **		
CPN	Percentage	100 (100, 100); 2.8 (1.1, 3.8)	82.5 (72.5, 90); 6.3 (0, 25)	0 (0, 0); 1.8 (1.2, 4.2)	
	*W*	27.5 **	14 **	63 **	
CBB	Percentage	98.8 (93.6, 100); 0.5 (0, 1.5)	50 (27.6, 71.8); 0 (0, 2.8)	0 (0, 0); 0.1 (0, 0.8)	0.8 (0.4, 1.3); 0.6 (0, 1)
	*W*	41.0 **	46.5 **	92.5 *	368 ^ns^

** *p* < 0.01, * 0.01 < *p* < 0.05, ns = not significant (*p* > 0.05).

**Table 2:** Mean (± SE) of life-history parameters of *Mesoneura rufonota* when reared on leaves from the five different tree species in the laboratory.

Tree Species	CCP	CBL	CMH	CPN	CBB
Larval survival (%)	84.33 ± 2.98a	45.00 ± 2.59b	3.67 ± 1.16c	10.33 ± 1.82c	10.67 ± 1.71c
Pupal survival (%)	88.88 ± 3.03a	54.11 ± 3.94b	0c	0c	0c
Larval developmental duration (d)	8.60 ± 0.06b	8.86 ± 0.08b	9.27 ± 0.30a	9.00 ± 0.15ab	8.97 ± 0.12ab
Pupal developmental duration (d)	7.25 ± 0.05b	7.73 ± 0.12a	-	-	-
Pupal weight (g)	0.23 ± 0.01a	0.20 ± 0.01b	0.16 ± 0.01c	0.19 ± 0.01b	0.19 ± 0.01b
Adult lifespan (d)	4.01 ± 0.05a	3.64 ± 0.10b	-	-	-

Means followed by the same letter within the same row are not significantly different (ANOVA, LSD test, *p* > 0.05).
